# Environmental contamination and risk factors for transmission of highly pathogenic avian influenza A(H5N1) to humans, Cambodia, 2006-2010

**DOI:** 10.1186/s12879-016-1950-z

**Published:** 2016-11-04

**Authors:** Sowath Ly, Sirenda Vong, Philippe Cavailler, Elizabeth Mumford, Channa Mey, Sareth Rith, Maria D. Van Kerkhove, San Sorn, Touch Sok, Arnaud Tarantola, Philippe Buchy

**Affiliations:** 1 Institut Pasteur in Cambodia, Phnom Penh, Cambodia; 2 Agence de Médecine Préventive, Ferney-Voltaire, France; 3 World Health Organization, Geneva, Switzerland; 4 Institut Pasteur, Paris, France; 5 National Veterinary Research Institute, Ministry of Agriculture Forestry and Fisheries, Phnom Penh, Cambodia; 6 Communicable Disease Control Department, Ministry of Health, Phnom Penh, Cambodia; 7 GSK Vaccines R&D, 150 Beach Road, 189720 Singapore, Singapore

**Keywords:** Environmental contamination, A (H5N1), Human seroprevalence, Risk factors

## Abstract

**Background:**

Highly pathogenic avian influenza A (H5N1) virus has been of public health concern since 2003. Probable risk factors for A(H5N1) transmission to human have been demonstrated in several studies or epidemiological reports. However, transmission patterns may differ according to demographic characteristics of the population and local practices. This article aggregates these data from three studies with data collected in the previous surveys in 2006 and 2007 to further examine the risks factors associated with presence of anti-A(H5) antibodies among villagers residing within outbreak areas.

**Methods:**

We aggregated 5-year data (2006–2010) from serology survey and matched case-control studies in Cambodia to further examine the risks factors associated with A(H5N1) infection among villagers in the outbreak areas.

**Results:**

Serotesting among villagers detected 35 (1.5 % [0–2.6]) positive cases suggesting recent exposure to A(H5N1) virus. Practices associated with A(H5N1) infection among all ages were: having poultry cage or nesting area under or adjacent to the house (OR: 6.7 [1.6–28.3]; *p* = 0.010) and transporting poultry to market (OR: 17.6 [1.6–193.7]; *p* = 0.019). Practices found as risk factors for the infection among age under 20 years were swimming/bathing in ponds also accessed by domestic poultry (OR: 4.6 [1.1–19.1]; *p* = 0.038). Association with consuming wild birds reached borderline significance (*p* = 0.066).

**Conclusion:**

Our results suggest that swimming/bathing in contaminated pond water and close contact with poultry may present a risk of A(H5N1) transmission to human.

## Background

Highly pathogenic avian influenza A (H5N1) virus has been of public health concern since 2003, particularly in south-east Asia and Egypt. The virus causes mass die-off in domestic poultry, occasionally infecting humans. As of 04 April 2016, 850 confirmed cases of A(H5N1) human infections worldwide with 449 deaths have been reported to the World Health Organization [1]. Probable risk factors for A(H5N1) transmission to human have been demonstrated in several studies or epidemiological reports [2]. However, transmission patterns may differ according to demographic characteristics of the population and practices leading to contact with poultry [3] and may be impacted by domestic waterfowl density and local geo-ecological settings [4].

In 2005, the first human case of A(H5N1) infection was reported in Cambodia, a country of approximately 15 million population. As of 04 April 2016, 56 confirmed cases have been identified, 37 of these fatal [1]. An investigation followed the identification of the first four human cases which found no evidence of mild or asymptomatic infection in the population [5]. Further investigations around the 5th, 6th and 7th human cases between 2006 and 2007, however, found evidence of mild or asymptomatic infections among villagers. These early studies explored the extent of contact with poultry and potential risk factors for A(H5N1) infections in humans, underscoring the possibility of an environmental source [6, 7]. As Cambodia continued to identify more A(H5N1) human cases during 2008–2010, we conducted three community investigations. This article aggregates these data from the three studies with data collected in the previous surveys in 2006 and 2007 to further examine the risks factors associated with presence of anti-A(H5) antibodies among villagers residing within the outbreak areas.

## Methods

### Documenting A(H5N1) circulation in poultry

Within one day after identification of a A(H5N1) human case, investigation teams from the National Veterinary Research Institute (NaVRI) and from Institut Pasteur in Cambodia (IPC) went to the village of the patient and conducted household visits within a one-kilometer radius of each case’s house. Household heads were interviewed using a standardized questionnaire, collecting information on poultry morbidity and mortality during the past 3 months including number of sick and dead poultry, dates of onset and symptoms. Venous blood, tracheal swab and cloacal swab specimens were taken from ≤10 randomly selected ducks in each household with duck flocks (chickens being extremely sensitive to A(H5N1) infection, no samples were taken from apparently healthy chickens). Blood samples were stored in 5 mL tubes without additive. Swab samples were put in tubes containing viral transport media (VTM) and shipped at temperature 4–8 °C to NaVRI laboratory in Phnom Penh within 48 h.

### A(H5N1) serological surveys in humans

During 2006–2010, six confirmed A(H5N1)-infected human cases were identified in five villages of five Cambodian provinces. Teams from IPC, the Cambodian Communicable Disease Control Department (Ministry of Health) and from the World Health Organization (WHO) conducted serological investigations in humans within a one-kilometer radius area approximately four to ten weeks after confirmation of the human A(H5N1) index cases. The objective was to interview and sample all villagers using a standardized questionnaire to collect demographic, poultry exposure and environmental exposure data for the three months preceding symptom onset in index cases. For children aged <12 years old, parents or guardians were interviewed. We used major social events such as Khmer or Lunar New Year, or Water Festival as temporal markers to help respondents recall past activities [3]. Informed consent was obtained and blood samples (5 mL) collected in tubes without additive were taken from all members of households and sent to IPC at temperature 4–8 °C within 48 h for serology testing.

As two human cases were detected in the same village successively in April 2007 and December 2009, blood specimens were collected twice from each subject with an interval of one month during the second investigation to draw laboratory conclusion on recent infection and to serologically rule out previous infection unrelated to the current event.

### Laboratory testing of poultry specimens

Poultry specimens were processed and tested at NaVRI laboratory as previously described [6]. Tracheal and cloacal swabs were inoculated into embryonated hen’s eggs. Allantoic fluid was tested to detect the presence of influenza virus by hemagglutination, followed by identification of H5 virus by hemagglutination inhibition assay (HIA). Positive (titer ≥1:16) specimens were sent to IPC for confirmation and virus subtyping using real-time reverse-transcriptase polymerase chain reaction (qRT-PCR) targeting the HA, M and NA genes [8]. Duck serum samples were tested using HIA method using reference H5 antigen provide by Office International des Epizooties (OIE). A poultry flock was considered infected with A(H5N1) virus if one swab sample or one serum sample tested positive.

### Serological testing in humans

Sera from the investigations conducted in 2006 were shipped to Influenza Division laboratories at the US Centers for Diseases Control and Prevention (Atlanta, USA) and sera from the four investigations in 2007, 2008, 2009 and 2010 were tested at IPC.

Sera from 2006 were tested by microneutralization (MN) assay and when the titer was ≥1:80, the result was confirmed by a Western blot assay, as recommended by WHO [6, 9].

Sera from 2007–2009 were screened using H5 hemagglutinin pseudotyped lentiviral particles (H5pp) expressing the hemagglutinin of a 2005 Cambodian H5N1 virus and those with a titer ≥1:20 were further tested by MN and hemagglutination inhibition (HI) assay using a 2007 Cambodian H5N1 strain [7, 10]. Antibody titers ≥1:80 in duplicate MN assays and ≥1:160 in duplicate HI test using horse red blood cells (HRBCs) were considered positive as per WHO recommendations [9].

Because the antigenic characteristics of the H5pp were no longer adapted to the strains detected after 2009, we modified the testing strategy. Beginning 2010, sera were initially screened by HI test using HRBCs and all samples with titers ≥1:10 were further tested by MN. In both assays, the viral strain isolated during the corresponding outbreak was used. Based on our findings suggesting that compared to severe cases, patients asymptomatically-infected developed lower antibody titers [11], in 2010 we slightly revised the criteria for positivity as follow: MN titer ≥1:80 associated with HI titer using HRBCs ≥1:80.

### Matched case-control studies

When seroprevalence surveys laboratory results became available, we conducted matched case-control studies by randomly selecting up to four A(H5N1)-seronegative subjects as controls for each case tested positive for anti-H5 antibodies. Controls were identified during the same investigation as cases and were matched to cases on age (+/- 3 years), gender, village of residence and households with suspected/confirmed A(H5N1) infection in poultry (chicken flock with >20 % mortality or ducks with confirmed A(H5N1). A standardized questionnaire was used to comprehensively document participants’ usual and recent activities in each affected village with regards to direct exposure to domestic poultry or wild birds (preparing food, children playing with poultry, husbandry practices), and indirect exposure with poultry in their living environment (house type, poultry cages/area, water source, ponds, rice field). Interviewers were not informed of respondents’ case-control status. Parents or guardians were interviewed if participants were <12 years old.

As we were unable to conduct in-depth interviews in 2009, exposure information was drawn from seroprevalence data documented for seropositive cases and randomly-selected, matched control subjects.

### Statistical analysis

Statistical analyses were conducted using Stata software (StataCorp, College Station, TX, USA). All tests were two-tailed and statistical significance level was set at *p* < 0.05. For statistical comparisons, we used Chi^2^ or Fisher Exact tests for proportions, Student test for means and Kruskal-Wallis test for medians. Conditional logistic regression models and Wald Chi^2^ test were used in bivariate and multivariate analyses to calculate maximum likelihood estimates for the matched odds ratios (OR) and 95 % confidence intervals (CI). All variables showing association at *p* < 0.20 with serology in bivariate analysis were included in the multivariate model using a backward process.

Three multivariate models were constructed with regards to the age of seropositive cases: (1) one model for all ages; (2) one for subjects aged less than 20 years; and (3) one model for subjects aged ≥20 years. Age and sex were taken into account in all multivariate models as possible confounding factors. All possible two-way interaction terms were tested separately on the final models and the likelihood ratio test was examined to find whether interaction terms significantly improved the model. Population attributable fractions (PAF) were computed for adjusted ORs associated with increased risk of infection.

## Results

### Documenting A(H5N1) circulation in poultry in villages where A(H5N1) human cases occurred

Between years 2006 − 2010, we surveyed 453 households within a 1-km radius from where the six index A(H5N1) human cases resided. The proportions of households with poultry morbidity or mortality ranged from 34 % − 82 % depending on the year. An above 20 % mortality in chickens was documented in 16 % − 75 % of chicken-rearing households. Among households whose ducks were tested, 30 % − 73 % had ≥1 duck which tested positive for anti-A(H5) antibody (Table 1). Additionally, we investigated one military training center by testing poultry raised there and identified H5-positive ducks.

**Table 1:** Mortality in chicken and serological results in ducks within outbreak areas, Cambodia, 2006-2010

Description	2006	2006	2007	2008	2009	2010
Village	Village 1	Village 2	Village 3	MTC	Village 3	Village 4
Province	KS	PV	KC	KD	KC	PV
HH visited	50	119	160	1	68	56
HH with poultry morbidity/mortality	17 (34 %)	93 (78 %)	80 (50 %)	1	30 (44 %)	46 (82 %)
HH raising chickens	44	107	111	1	50	53
HH with chicken mortality >20 %	7 (16 %)	80 (75 %)	67 (60 %)	1	18 (36 %)	32 (60 %)
HH raising ducks	23	59	13	1	11	29
HH where blood samples were collected from ducks	19	33	9	1	10	4
HH with positive ducks for anti-A(H5) antibody	10 (52 %)	24 (73 %)	3 (33 %)	1	3 (30 %)	2 (50 %)
Poultry die-off Approximate start date	07 Feb 2006	15 Jan 2006	01 Feb 2007	15 Oct 2008	05 Dec 2009	25 Jan 2010
Date of symptom onset in human A(H5N1) case	14 Mar 2006	29 Mar 2006	2 Apr 2007	28 Nov 2008	11 Dec 2009	13 Apr 2010
Duration from poultry die-off to clinical onset in human case	35 days	73 days	60 days	44 days	6 days	78 days

*KD* Kandal, *KS* Kampong Speu, *PV* Prey Veng, *MTC* Military training center, *HH* Households

### A(H5N1) serological surveys in humans

During 2006–2010, the six serosurveys around identified human cases among people living in four villages and a military training center located in four Cambodian provinces. A total of 2,758 participants were included, interviewed and sampled. Two surveys were conducted in Village 3, Kampong Cham province, following A(H5N1) confirmation in one human case in 2007 and another case in 2009 (Table 1).

Only data from 2006, 2007, 2009 and 2010 were combined for analyses (*n* = 2,364). Among the 2,364 participants, 1,078 (45.6 %) were male, 1,188 (50.3 %) were farmers and 1,007 (42.6 %) were children or school students. Median age was 20 years (range: 3.6 months-99 years; IQR: 3 months-87 years). Serology testing detected 35 (1.5 %) subjects with positive results suggesting recent exposure to A(H5N1) virus. The prevalence for specific study years were 1.0 % (7/674) in 2006, 2.6 % (18/700) in 2007, and 1.6 % (10/624) in 2009. No participant tested positive in 2010. Of the 35 subjects positive for anti-A(H5) antibodies, 19 (54.3 %) were male, 15 (42.9 %) were farmers, 19 (54.3 %) were children or school students and the median age was 16 years (range: 3–77; IQR: 4–70) (Table 2). Twenty-one (60.0 %) of positive subjects were aged less than 20 and 6 (17.1 %) were aged between 20 and 39 years. In 2006, all positive subjects were aged <20 years while this age group was less represented at 61.1 % in 2007 and 30.0 % in 2009 (*p* = 0.015).

**Table 2:** Characteristics of participants found positive for anti-A(H5) antibodies, Cambodia, 2006−2010

Year of investigation	All *n* (%)	2006 *n* (%)	2007 *n* (%)	2009 *n* (%)	2010 *n* (%)	*p* value
Participants	2,364	674	700	624	366	
Positive for anti-A(H5)	35 (1.5)	7 (1.0)	18 (2.6)	10 (1.6)	0	
Positive participants						
Gender - male	19 (54.3)	6 (85.7)	8 (44.4)	5 (50.0)	-	0.179
Age (years)						
Median (min. - max.)	16 (3-77)	12 (4-18)	14.5 (3-77)	26.5 (5-70)	-	0.239
Means	24.2	11.4	25.7	30.6	-	
Age group						
0−4 years	4 (11.4)	2 (28.6)	2 (11.1)	0	-	0.245
5−19 years	17 (48.6)	5 (71.4)	9 (50.0)	3 (30.0)	-	
20−39 years	6 (17.1)	0	2 (11.1)	4 (40.0)	-	
40−59 years	3 (8.6)	0	2 (11.1)	1 (10.0)	-	
>59 years	5 (14.3)	0	3 (16.7)	2 (20.0)	-	
Age under 20	21 (60.0)	7 (100)	11 (61.1)	3 (30.0)	-	0.015
Occupation						
Farmer	15 (42.9)	1 (14.3)	7 (38.9)	7 (70.0)	-	0.123
Worker - rubber plantation	1 (2.9)	0	1 (5.6)	0	-	
Student or child	19 (54.3)	6 (85.7)	10 (55.6)	3 (30.0)	-	

Participants of 2008 survey were excluded from the analysis (Kandal province, *n* = 394, military, mainly male aged 18 − 35 years old). None of them tested positive for anti-A(H5) antibodies

Among study participants at a military center in Kandal province in 2008 (*n* = 394), most were military trainees (80.7 %), male (87.3 %), aged between 18 and 35 years old (76.4 %), and trainees who stayed at the center for at least 3 months. These participants all tested negative.

### Factors associated with A(H5N1) infections to human

In total 35 cases and 115 matched control subjects were enrolled in the case-control studies (pooled data for 2006, 2007 and 2009 excluding military recruits). Bivariate analysis using conditional logistic regression for matched case-control analysis identified variables significantly associated with seropositivity at a <20 % level (Table 3), which were included in the multivariate analyses.

**Table 3:** Bivariate analysis of factors associated with A(H5N1) virus infection in humans, data from matched case-control studies in 2006, 2007, 2009, Cambodia

Characteristic	Control	Case	OR	95 % CI	*p* value (*)
	n/N (%)	n/N (%)			
All ages	(*n* = 115)	(*n* = 35)			
Poultry cage or nest located under or adjacent to the house	36/68 (52.9)	14/18 (77.8)	3.2	0.9−11.0	0.060
Having a pond in the house yard	49/115 (42.6)	10/35 (28.6)	0.4	0.1−1.2	0.109
Swimming or bathing in ponds accessed by poultry	35/115 (30.4)	14/35 (40.0)	2.1	0.9−4.8	0.099
Swimming or bathing in ponds visited by domestic and wild poultry	19/65 (29.2)	8/17 (47.1)	2.2	0.7−6.8	0.157
Feeding poultry	84/115 (73.0)	20/35 (57.1)	0.4	0.2−1.0	0.055
Collecting poultry feces for use as fertilizer	22/91 (24.2)	3/28 (10.7)	0.4	0.1−1.4	0.141
Presence of SD animals in the house or house yard	59/92 (64.1)	11/25 (44.0)	0.4	0.2−1.1	0.083
Presence of SD poultry in neighboring households	61/90 (67.8)	12/28 (42.9)	0.4	0.1−0.9	0.023
Handling SD with bare hands	64/115 (55.7)	15/35 (42.9)	0.6	0.3−1.3	0.181
Preparing SD poultry for food	34/115 (29.6)	7/35 (20.0)	0.5	0.1−1.4	0.178
Eating food prepared from SD poultry	57/91 (62.6)	10/28 (35.7)	0.3	0.1−0.8	0.015
Handling SD wild birds	8/92 (8.7)	5/25 (20.0)	2.4	0.8−7.8	0.131
Transporting poultry to trade	2/92 (2.2)	3/25 (12.0)	6.0	1.0−35.9	0.050
Presence of a household member with flu-like illness during the outbreak	60/115 (52.2)	15/35 (42.9)	0.5	0.2−1.2	0.108
Providing care for the case during his/her illness or for the case's corpse	21/92 (22.8)	2/25 (8.0)	0.3	0.1−1.4	0.120
Age <20 years old	(*n* = 77)	(*n* = 22)			
Poultry cage or nest located under or adjacent to the house	23/43 (53.5)	9/11 (81.8)	4.1	0.8−21.7	0.095
Having a pond in the house yard	37/77 (48.1)	7/22 (31.8)	0.1	0.02−1.2	0.076
Swimming or bathing in ponds also accessed by poultry	28/77 (36.4)	12/22 (54.6)	2.6	0.9−7.3	0.065
Swimming or bathing in ponds also accessed by wild birds	16/42 (38.1)	7/11 (63.6)	2.9	0.8−11.4	0.119
Feeding poultry	59/77 (76.6)	12/22 (54.6)	0.2	0.05−0.8	0.027
Presence of SD poultry in neighboring households	33/53 (62.3)	6/15 (40.0)	0.4	0.1−1.4	0.145
Handling SD with bare hands	44/77 (57.1)	9/22 (40.9)	0.5	0.2−1.4	0.161
Eating food prepared from SD poultry	31/53 (58.5)	5/15 (33.3)	0.3	0.08−1.2	0.096
Handling SD wild birds	6/67 (9.0)	5/18 (27.8)	3.3	0.9−11.7	0.059
Eating food prepared from wild birds	18/67 (26.9)	9/18 (50.0)	3.5	0.98−12.7	0.054
Age ≥20 years old	(*n* = 38)	(*n* = 13)			
Gathering and placing poultry in their cages or area	15/38 (39.5)	9/13 (69.2)	3.0	0.8−11.6	0.104
Presence of SD animals in the house of house yard	16/38 (64.0)	2/13 (28.6)	0.2	0.02−1.8	0.142
Presence of SD poultry in neighboring households	28/37 (75.7)	6/13 (46.2)	0.3	0.07−1.2	0.079
Eating food prepared from SD poultry	26/38 (68.4)	5/13 (38.5)	0.2	0.05−1.2	0.075

*OR* odd ratio, bivariate analysis using conditional logistic regression model, *CI* confidence interval, *SD* sick or died from disease, Case: subject tested positive for anti-H5 antibodies; (*) only exposure variables included in multivariate analysis (significant level *p* < 0.2) are shown

Among subjects of all ages, the following exposures were more frequently reported by cases positive by A(H5N1) serology compared to matched controls: having a poultry cage or nesting area located under or right next to the house (77.8 % vs 52.9 %; *p* = 0.060); swimming in ponds also accessed by poultry (40 % vs 30.4 %; *p* = 0.099); swimming in ponds also accessed by wild birds (47.1 % vs 29.2 %; *p* = 0.157); handling sick/dead wild birds (20.0 % vs. 8.7 %; *p* = 0.131); and transporting poultry to market (12.0 % vs. 2.2 %; *p* = 0.050).

In the subgroup of 22 cases aged <20 and their 77 matched control subjects, the following practices were more frequently reported among seropositive cases: having a domestic poultry cage or nesting area located under or adjacent to the subject’s house (81.8 % vs 53.5 %; *p* = 0.095); swimming in ponds frequented by domestic poultry (54.6 % vs 36.4 %; *p* = 0.065) or in ponds visited by domestic and wild poultry (63.6 % vs 38.1 %; *p* = 0.119); handling sick/dead wild birds with bare hands (27.8 % vs 9.0 %; *p* = 0.059); and eating wild birds as food (50.0 % vs. 26.9 %; *p* = 0.054).

Among the 13 cases aged ≥20 years and their 38 control subjects, gathering and placing domestic poultry in the cages or designated poultry area was somewhat more frequent among seropositive cases (69.2 % vs. 39.5 %; *p* = 0.104).

Results from three conditional logistic regression models are presented in Table 4. Among subjects of all ages (35 cases and 115 controls), practices that appeared to be risk factors for A(H5N1) infection in humans after adjusting for the other variables were: having a domestic poultry cage or nesting area located under or adjacent to the subject’s house (matched OR: 6.7 [range 1.6–28.3]; *p* = 0.010; PAF = 66.2 %) and transporting domestic poultry to market (adjusted OR: 17.6 [range 1.6–193.7]; *p* = 0.019; PAF = 11.0 %). Factors that were associated with or bordered on a lower risk of transmission were: presence of a pond in the house yard (adjusted OR: 0.2 [range 0.06–0.8]; *p* = 0.027); presence of sick animals or animal carcass died of illness in the house yard (adjusted OR: 0.2 [range 0.04–0.6], *p* = 0.008); and providing care for the A(H5N1) index case during the illness or the case's corpse (adjusted OR: 0.17 [range 0.03–0.97]; *p* = 0.046).

**Table 4:** Multivariate analysis of risk factors associated with A(H5N1) virus infection in various age groups, data from seroprevalence surveys in 2006, 2007 and 2009, Cambodia

Characteristic	OR	95 % CI	*p* value	Prevalence among cases (%)	PAF (%)
All ages (35 cases and 115 control subjects)					
Poultry cage or place located under or attached to the house	6.7	1.6-28.3	0.010	77.8	66.2
Presence of a pond in the house yard	0.2	0.06-0.8	0.027	28.6	-
Presence of sick animals or animal that died from disease in the house yard	0.2	0.04-0.6	0.008	44.0	-
Transporting poultry to trade	17.6	1.6-193.7	0.019	12.0	11.0
Providing care for the case during the illness or for the case's corpse	0.17	0.03-0.97	0.046	80.0	-
Age under 20 years old (22 cases and 77 control subjects)					
Swimming or bathing in ponds also accessed by poultry	4.6	1.1-19.1	0.038	54.6	42.7
Feeding poultry	0.2	0.03-0.8	0.030	54.6	-
Eating food prepared from sick poultry or poultry that died from disease	0.2	0.02-1.0	0.051	33.3	-
Eating wild birds	4.9	0.9-26.8	0.066	50.0	39.8
Age ≥20 years old (13 cases and 38 control subjects)					
Gathering and placing poultry in their cages or nests	4.3	0.9-21.5	0.072	69.2	53.1
Eating food prepared from sick poultry or poultry that died from disease	0.1	0.02-1.0	0.055	38.5	-

*OR* odd ratio, multivariate analysis using conditional logistic regression model, *CI* confidence interval, *PAF* Population Attributable Fraction

In subjects aged <20 years (22 cases and 77 controls), practices found to be risk factors for A(H5N1) infection were swimming in ponds accessed by domestic poultry (adjusted OR: 4.6 [range 1.1–19.1]; *p* = 0.038; PAF = 42.7 %). Eating wild birds (adjusted OR: 4.9 [range 0.9–26.8]; *p* = 0.066; PAF = 39.8 %) bordered on statistical significance. Practices associated with a lower risk was routinely feeding household poultry (adjusted OR 0.2; [range 0.03–0.8]; *p* = 0.03).

In subjects aged ≥20 years (13 cases and 38 controls), gathering and placing poultry in their cage/nesting area bordered on statistical association with A(H5N1) infection (adjusted OR: 4.3 [range 0.9–21.5]; *p* = 0.072; PAF = 53.1 %) while eating sick/dead poultry tended to be protective (adjusted OR: 0.1 [range 0.021–1.0]; *p* = 0.055).

## Discussion

Four consecutive serological surveys in humans conducted around identified cases during four of five years (2006–2010) by the same Khmer-speaking Cambodian field epidemiology team experienced in administering the same standardized questionnaire and collecting samples documented a low prevalence of A(H5N1) antibodies. These samples were analyzed by a reference diagnosis laboratory virology for A(H5N1). Although reference A(H5N1) diagnostic procedures improved with time, seroprevalence remained comparable across the years and even receded. At 1.5 % (ranging from 1.0 % in 2006 − 2.6 % in 2007), this low prevalence is in line with findings among populations not involved in poultry culling or sales, mainly in rural settings [1, 12–17].

The nested matched case-control study was undertaken to identify risk factors for transmission among those with evidence of recent infection by A(H5N1).

Exposure to A(H5N1) translates into infection risks at any age including young adults. When considering all age groups, having a poultry cage directly below the floor of stilt houses or appended to non-stilted houses was a significant independent risk factor. This confirms findings from studies in China [18] and Thailand [19] showing that living in close proximity to dead or sick poultry shedding virus or poultry increases infection risk. Transporting poultry to markets or collection sites was also an independent factor when all age groups were considered.

Childhood activities such as swimming in village ponds was significantly associated with recent A(H5N1) infection (OR: 4.6; *p* = 0.038), confirming data from previous studies in Cambodia [6, 7]. In villages located far from the river, the alternative water source and water storage can be a small pond for washing/bathing, drinking/cooking, gardening and sometimes raising fish or ducks. Ducks that can potentially be infected by A(H5N1) virus have access to these communal ponds and may defecate/shed large quantities of virus in pond water [20–22]. A(H5N1) infections in two children in Vietnam may be linked to swimming/bathing in canal water also accessed by ducks [23]. Testing of environmental specimens taken from the ponds in A(H5N1)-affected area detected viral RNA in several samples including mud, water and aquatic plants [24, 25]. Experiments have shown that infectious particles of A(H5N1) virus may survive in water and fauna for days even at high temperature, demonstrating the potential threat of contaminated ponds [26]. Moreover, studies in Vietnam [27] and Thailand [28] showed that documented human cases of A(H5N1) infection were significantly associated with lack of indoor water sources.

Another identified activity common among children in the Cambodian countryside is preparing and consuming wild birds such as sparrows and cranes. The association of consuming wild birds with markers of recent A(H5N1) infection bordered on significance (OR: 4.9; *p* = 0.066). These birds are traditionally shot down or trapped and grilled over a small fire in the fields. Often, the deepmost internal parts of the bird are not fully cooked. Human cases of infection with A(H5N1) virus following direct exposure to dead wild birds were reported in Azerbaijan [29] and consuming uncooked duck blood has been suggested as a source of infection in some cases in Vietnam [30]. It has also been experimentally shown that small sparrows could theoretically be infected after contact with infected poultry and serological evidence of influenza contamination of these birds has been found in Cambodia [31].

In Cambodia, gathering or placing poultry in cages is usually done by older persons (aged 20 or above). The association of this practice with biological signs of recent infection bordered on significance (OR: 4.3; *p* = 0.072). The link between this activity and A(H5N1) infection was also well documented in Egypt [32].

Some factors were statistically associated with a lower risk of having markers of recent A(H5N1) infection. In subjects of all age, having a (private) pond in the house yard or presence of sick or dead poultry in the house yard were protective factors (OR: 0.2, *p* = 0.027 and OR: 0.2, *p* = 0.008 respectively). This is not paradoxical when the Cambodian context is taken into account. Having a pond in the yard entails a lesser probability of interacting with sick poultry in these private, well-maintained and protected ponds than in the communal ponds described above. Additionally, it provides better access to water and perhaps better personal hygiene practices such as hand washing after handling sick poultry. Having sick or dead poultry in the house yard is not in itself a good proxy for A(H5N1) circulation among poultry in Cambodia, where many co-circulating pathogens cause poultry death [33]. Furthermore, households in which poultry deaths occurred may be more prone to observe prevention messages and implement preventive measures.

The latter was equally true of “providing care for an identified human case of A(H5N1)”, also found to be protective. Despite one human-to-human transmission event in Thailand, available epidemiological data show that A(H5N1) transmission to those providing care is extremely rare [34, 35]. Furthermore, our detailed interviews showed that most study subjects who declared they provided care actually did little more than visit the human cases, without close or prolonged contact.

Paradoxically, eating food prepared from sick or dead poultry tended to be a protective factor. Although consuming well-cooked meat of infected poultry is well documented as posing little or no risk [36], the fact that this bordered on significance and appeared in independent analyses for both age groups but not in all-age analysis suggest that this is an artefact.

Among subjects aged below 20, feeding poultry was associated with a lower risk of recent A(H5N1) infection. Feeding poultry was understood in these surveys as a habitual activity of feeding live poultry. As backyard flocks in our settings were overwhelmingly constituted of chickens and not ducks, this activity was likely interrupted by signs of disease in the poultry and did not incur exposure to A(H5N1).

Our findings are subject to bias and limitations. Firstly, factors investigated were suggested by the literature and profound knowledge of the Cambodian setting but some additional factors may have been missed, such as individual host genetic factors [36]. However, this case-control study with cases matched on age (±3 years) and gender and the presence of A(H5N1) detected in at least some household poultry in the same villages naturally adjusts for many unidentified but possibly linked factors, such as host genetics in these Cambodian villages with low ethnic intra-village diversity.

Secondly, technical limitations may have biased our findings. During the five-year period, progressively improved assays may alter the year-to-year comparability of seroprevalence findings and the characteristics of cases. This improvement of diagnostic methods is to be expected from a reference laboratory and is unavoidable, but using the most recent reference strains improved detection in cases and therefore increased the sensitivity in our detection of factors associated with higher risk. In addition, if the screening approach differed over the time, the criteria for positivity essentially only slightly changed in 2010 after one of our study suggested that asymptomatically-infected individuals were expected to have lower antibody titers than severe human H5N1 cases. Thirdly, A(H5N1) antibodies decay could theoretically be associated with false-negative findings in study subjects [11]. Our seroprevalence surveys, however, were conducted within four weeks of the detection of index cases and poultry die-off.

Finally, our study is based on a limited number of surveys and cases. Although low power may explain borderline significance of some findings, a total of 35 cases and 115 controls provides adequate power to detect factors with a strong association, which are the most relevant in terms of public health and prevention. Furthermore, the fact that these were documented across several years in different villages reduces the risk of documenting risk or protective factors in a single setting at a single moment in time. Investigators who interviewed and sampled participants were *de facto* blind to subsequent serological results, adding to the reliability of the study findings.

## Conclusion

Our findings from surveys conducted over several years in several different villages identifying proximity to poultry cages and swimming in communal ponds accessed by humans and poultry can reliably be extrapolated to the general Cambodian setting. Additionally, these surveys were conducted in the general population, not in high-risk groups such as veterinarians or poultry workers.

Findings were communicated to public health authorities who have since reinforced prevention messages. The identification of possible increased risks of A(H5N1) infection linked with hunting and consuming wild birds in Cambodia warrants further and careful study. Additional work is being conducted on documenting possible genetic polymorphisms associated with increased vulnerability or resistance to A(H5N1) infection in humans [34].

## Abbreviations

HI: Hemagglutination inhibition
HIA: Hemagglutination inhibition assay
HRBCs: Horse red blood cells
IPC: Institut Pasteur in Cambodia
MN: Microneutralization
NaVRI: National veterinary research institute
OIE: Office International des Epizooties
rRT -PCR: Real-time reverse-transcriptase polymerase chain reaction
VTM: Viral transport medium
WHO: World Health Organization
